# Electrochemical reduction of nitrobenzene via redox-mediated chronoamperometry

**DOI:** 10.1016/j.xpro.2022.101817

**Published:** 2022-11-04

**Authors:** Athanasios D. Stergiou, Daniel H. Broadhurst, Mark D. Symes

**Affiliations:** 1 WestCHEM, School of Chemistry, University of Glasgow, University Avenue, Glasgow G12 8QQ, UK

**Keywords:** NMR, Chemistry, Material sciences

## Abstract

Anilines are important feedstocks for pharmaceuticals, dyes, and other materials, but traditional approaches to their syntheses usually lack selectivity and environmental sustainability. Here, we describe the selective reduction of nitrobenzene to aniline under mild conditions, using water as the ultimate source of the required protons and electrons. We describe the electrochemical cell assembly, and detail steps for electrochemical reduction followed by organic extraction and analysis of the extracts using NMR.

For complete details on the use and execution of this protocol, please refer to Stergiou and Symes (2022a).

## Highlights

• A protocol for the electrochemical reduction of nitrobenzene to aniline
• The approach uses a polyoxometalate redox mediator to ensure high selectivity
• The electrochemical setup and parameters are described in detail

## Abstract (editor highlights)

Publisher’s note: Undertaking any experimental protocol requires adherence to local institutional guidelines for laboratory safety and ethics.

## Graphical abstract

**Figure:**
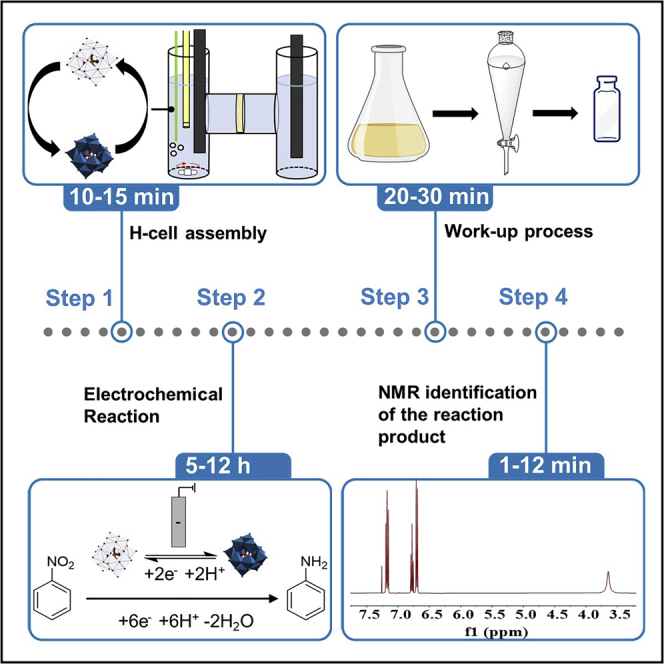

## Before you begin

Aromatic amines (anilines) are essential building blocks, extensively used in the industrial sector for the synthesis of pharmaceuticals, plastics, dyes, and agrochemicals (Kadam and Tilve, 2015; Jurado-Sanchez et al., 2013). A typical example of such a compound is the substituted nitrobenzene *p*-aminophenol, which is used as a precursor in the synthesis of one of the most widely produced pharmaceuticals, paracetamol (Ellis, 2002). At present, the most widespread route for the synthesis of these compounds is through the reduction of their corresponding nitrobenzenes (Travis, 2009). However, these transformations require the use of high temperatures (about 300°C) and pressures (5 bar), the use of extremely flammable hydrogen gas as well as expensive metal-based catalysts (Pt, Ni, Pd, or Cu) (Formenti et al., 2019). These routes are usually very effective for the reduction of the simple un-substituted nitrobenzene but their selectivity for the production of functionalized nitroarenes is somewhat more variable (Formenti et al., 2019). A prime example of such suboptimal selectivity is the transformation of *o-*nitroiodobenzene to its corresponding aniline derivative, where the halogen is readily cleaved from the benzene ring causing the formation of side products (Pietrowski, 2012; Baumeister et al., 1991). To tackle the selectivity issue in the reduction of substituted nitrobenzenes, more recent approaches have focused on the use of a different hydrogenation agent (other than H_2_) and less harsh reaction conditions (Gladiali and Alberico, 2006; Samec et al., 2006). One alternative is the use of dissolved hydrogenation agents such as hydrazine and formate in the presence of metal catalysts (Layek et al., 2012; Doherty et al., 2019; Garcia-Verdugo et al., 2006). The use of these reagents, however, raises some questions concerning sustainability as these compounds are toxic and are irreversibly consumed during the hydrogenation process.

Electrochemistry on the other hand can offer a sustainable alternative to various organic transformations currently used in industry (Stergiou and Symes, 2022b). Regarding the reduction of nitrobenzenes to anilines, electrochemistry can offer a sustainable alternative where the protons and electrons required can originate from the electrolysis of water. To date, however, electrochemical approaches to the reduction of nitrobenzenes to anilines still lack selectivity in most cases or suffer from competing reactions, especially the hydrogen evolution reaction which decreases the Faradaic yield of the process. Marquez and Pletcher conducted seminal work on the direct electrochemical reaction of nitrobenzene at the electrode surface, the main result of which was the identification of procedures that yielded primarily the *p-*aminophenol derivatives, rather than anilines (Marquez and Pletcher, 1980). More recent approaches have utilized metal-based electrocatalysts to enhance the selectivity of these processes towards anilines (Chong et al., 2020; Zhao et al., 2020; Daems et al., 2018; Li et al., 2007; Zhang et al., 2014). However, to the best of our knowledge, none of these aforementioned processes possesses all of the key elements (high aniline selectivity, high nitrobenzene conversion, high Faradaic efficiency, and mild process conditions) required by an effective and general electrochemical process for nitrobenzene reduction.

The protocol below describes the specific steps for such a general electrochemical process for reducing nitrobenzene to aniline. We have also used this protocol to reduce a range of substituted nitrobenzenes to their corresponding anilines, as detailed in Stergiou and Symes (2022a). The electrocatalytic system consists of a custom-made H-cell, electrolyte solutions (containing phosphotungstic acid as a redox mediator), and electrodes as described below. In this example, a BioLogic SP-150 potentiostat is employed to perform the electrochemical experiment. Other potentiostats, and indeed power supplies of less sophisticated operation, can also be used to produce the same results.

If needed, prepare the electrolyte solutions. A description of each of the required electrolytes is given below.

### Preparing 1 M sulfuric acid solution

**Timing: 15 min**

1. Concentrated sulfuric acid (95%) is diluted with deionized water to yield a 1 M sulfuric acid aqueous solution.
  a. First, add 100 mL of deionized water to a 1 L volumetric flask.
  b. Next, slowly add 56.42 mL (98.08 g or 1 mol) of sulfuric acid to the volumetric flask.
  c. Next, top-up the flask to 1 L with deionized water.
  d. Finally, stir the aqueous solution on a stirring plate for around 5 min.

### Preparing 1 M phosphoric acid solution

**Timing: 15 min**

2. Concentrated phosphoric acid (85%) is diluted with deionized water to yield a 1 M phosphoric acid aqueous solution.
  a. First, add around 100 mL of deionized water to a 1 L volumetric flask.
  b. Next, slowly add 68.42 mL (98.00 g or 1 mol) of phosphoric acid to the volumetric flask.
  c. Next, top-up the flask to 1 L with deionized water.
  d. Finally, stir the aqueous solution on a stirring plate for around 5 min.

### Preparing 1 M sodium hydroxide solution

**Timing: 15 min**

3. Sodium hydroxide is dissolved and diluted in deionized water to yield a 1 M sodium hydroxide solution.
  a. First, add around 500 mL of deionized water to a 1 L volumetric flask.
  b. Add a magnetic stirring bar into the flask and place it on a stirring plate.
  c. Next, slowly add 40 g of sodium hydroxide pellets into the volumetric flask.
  d. Next, top-up the flask to 1 L with deionized water.
  e. Finally, stir the solution until all the NaOH pellets are dissolved.

### Activating the Nafion proton-exchange membrane

**Timing: 24 h**

**Figure 1:**
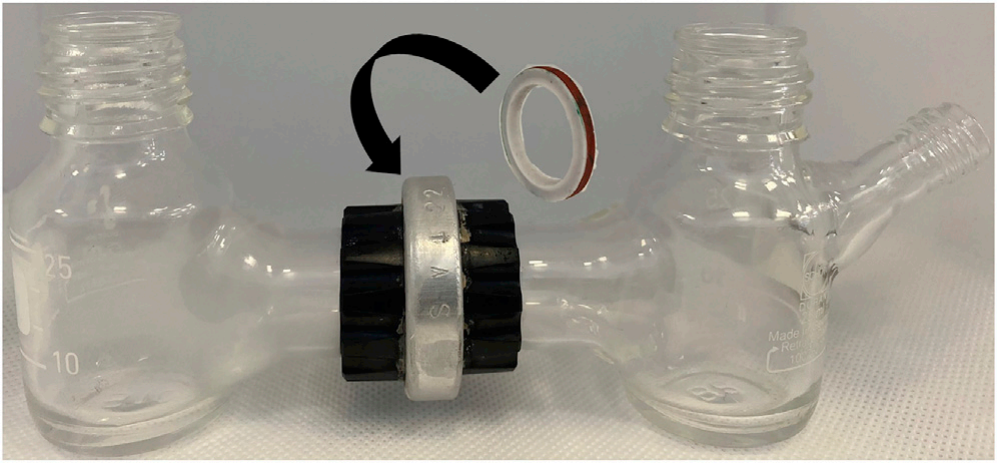
Custom-made two-compartment electrochemical H-cell
  b. Place the Nafion strips into a clean, beaker containing 1 M sulfuric acid and ensure the strips are completely submerged. They must remain submerged for a minimum of 24 h before moving on to the next step.
  c. After cell assembly (see below), use tweezers to remove a Nafion strip from the beaker and place it onto a clean, dry tissue and gently dab the strip to dry it. Using the sealing ring as a guide, carefully cut the membrane to the correct size.

4. Nafion-1110 was used in this case which requires activation in acidic media prior to use.
  a. Cut the as-purchased Nafion using a pair of scissors into strips with a width slightly greater than that of the sealing ring (20 mm width and 3–4 cm length) that separates the two compartments (see cell design in Figure 1). This is to minimize the amount of Nafion waste.

### Electrochemical cell (H-cell) assembly

**Timing: 1 h**

**Figure 2:**
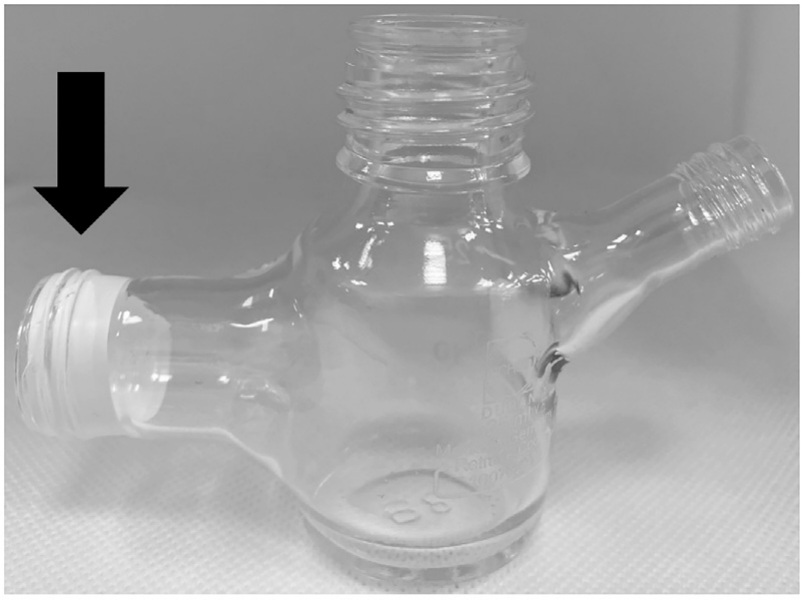
Teflon tape used at the threading of the compartment joint to prevent leakage
  c. Insert the sealing ring and then join the male ends together with the threaded H-cell connector (item D in Figure 3).

5. A two-compartment H-cell equipped with a proton-exchange membrane will be required for the electro-reduction process. In this case, a custom-made two-compartment cell (see Figure 1) was used to perform the reduction of nitrobenzene. Each side has a 35 mL capacity. A circular sealing ring is placed between the two compartments.
  a. The cell must be cleaned thoroughly with deionized water and acetone and dried completely before starting.
  b. Construct the cell so that it is leak-free. To ensure that there is no leakage in this example, Teflon tape is wrapped around the male-end threading of each compartment joint (see Figure 2).

**Figure 3:**
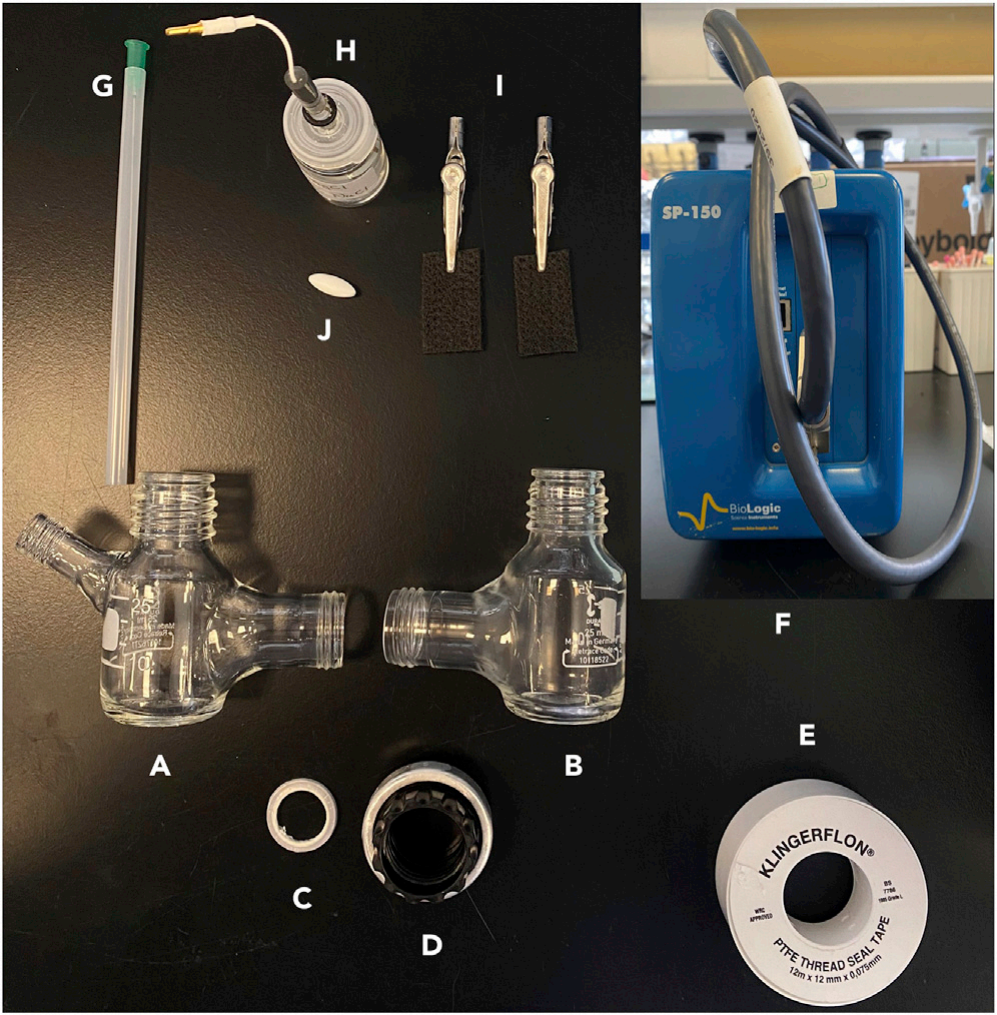
Overall diagram of the components of the electrochemical reaction, with parts labeled as follows (A) Working side of the H-cell. (B) Counter side of the H-cell. (C) Sealing ring with Nafion membrane. (D) H-cell connector. (E) Teflon tape. (F) BioLogic SP-150 potentiostat. (G) Syringe needle for N_2_ purge. (H) Ag/AgCl reference electrode. (I) Working and counter electrodes. (J) Stirring bar.
  d. Test the cell by filling each compartment with deionized water and check for any leaks over a period of about 30 min prior to use (see troubleshooting 1). Then empty the water from the cell.
6. Preparation of electrodes.
  a. The working and counter electrodes used are both strips of carbon felt. The counter electrode should be at least of equal size, if not larger, than the working electrode. In this case, cut two rectangles of equal size, 1.8 × 2 cm (geometric surface area), using a pair of scissors.
  b. Use stainless steel crocodile clips to connect the electrodes to the potentiostat.
  c. The reference electrode of choice was Ag/AgCl (3 M NaCl), which when not in use was stored in a 3 M NaCl solution.

An overall diagram of all the components used in the electrochemical setup is presented in Figure 3.

## Key resources table

**Table:** 

REAGENT or RESOURCE	SOURCE	IDENTIFIER
**Chemicals, peptides, and recombinant proteins**		

Phosphotungstic acid hydrate	Sigma-Aldrich	CAS#12501-23-4
Nitrobenzene	Sigma-Aldrich	CAS#98-95-3
Sodium hydroxide	Sigma-Aldrich	CAS#1310-73-2
Chloroform	Fisher Scientific	CAS#67-66-3
Sulfuric acid	Fisher Scientific	CAS#7664-93-9
Nafion N-1110	Fuel Cell Store	Cat#590149-1
Phosphoric acid	Alfa Aesar	CAS#7664-38-2

**Software and algorithms**		

Origin 2020	OriginLab	https://www.originlab.com
Mestrenova	Mestrelab Research	https://mestrelab.com/
EC-Lab	BioLogic	https://www.biologic.net/

**Other**		

Nuclear Magnetic Resonance (NMR)	Bruker, UK	400 MHz
Carbon felt, 3.18 mm thick, 99%	Alfa Aesar	Cat#43199
Silver/Silver chloride reference electrode	Alvatek	Cat#MF-2052
Stainless steel crocodile clips	RS Components	Cat#169-1838
Two-compartment (H-cell)	Custom made	–
Sealing ring and cell connector	Duran Wheaton Kimble Life Sciences	Item Code# 701-12
BioLogic SP-150	BioLogic	https://www.biologic.net/products/sp-150/
Deuterated chloroform, 99.8% (CDCl_3_)	Cambridge Isotope Laboratories, Inc.	Cat# DLM-7-100MS
IKA RCT digital stirring plate	IKA	https://www.ika.com/en/Products-Lab-Eq/Magnetic-Stirrers-Hot-Plate-Lab-Mixer-Stirrer-Blender-csp-188/IKA-Plate-(RCT-digital)-cpdt-25004601/
Rotary evaporator	BUCHI	https://www.buchi.com/

**CRITICAL:** Phosphoric acid is corrosive and an irritant. It can cause skin burns and eye damage and therefore it needs to be handled carefully. When handling phosphoric acid, gloves, safety specs, and a lab coat should be worn.

**CRITICAL:** Phosphotungstic acid is corrosive, an irritant, and toxic to aquatic life. It can cause severe skin burns and eye damage. When handling phosphotungstic acid, gloves, safety specs, and a lab coat should be worn.

**CRITICAL:** Chloroform is toxic and a suspected carcinogen. It can cause skin and eye irritation and should be used in a fume cupboard wearing gloves, safety specs, and a lab coat.

**CRITICAL:** Nitrobenzene is toxic and a suspected carcinogen. It should only be used in a fume cupboard wearing gloves, safety specs, and a lab coat.

**CRITICAL:** Aniline is toxic, a suspected carcinogenic, corrosive, and toxic to aquatic life. It should be used in a fume cupboard and handled wearing gloves, safety specs, and a lab coat.

***Alternatives:*** A wide range of potentiostats can perform chronoamperometry (sometimes also called bulk electrolysis with coulometry), and any such machine can be used to perform the reduction of nitrobenzene.

***Alternatives:*** Moreover, different NMR machines with different magnetic field strengths and any NMR solvent that can dissolve aniline can be used.

***Alternatives:*** Any solvent that is immiscible with water and can dissolve aniline (such as diethyl ether) can be used during the organic extraction stage.

## Step-by-step method details

### Electrolysis

**Timing: 1 h set-up plus 5–12 h run-time depending on the starting material**

**Figure 4:**
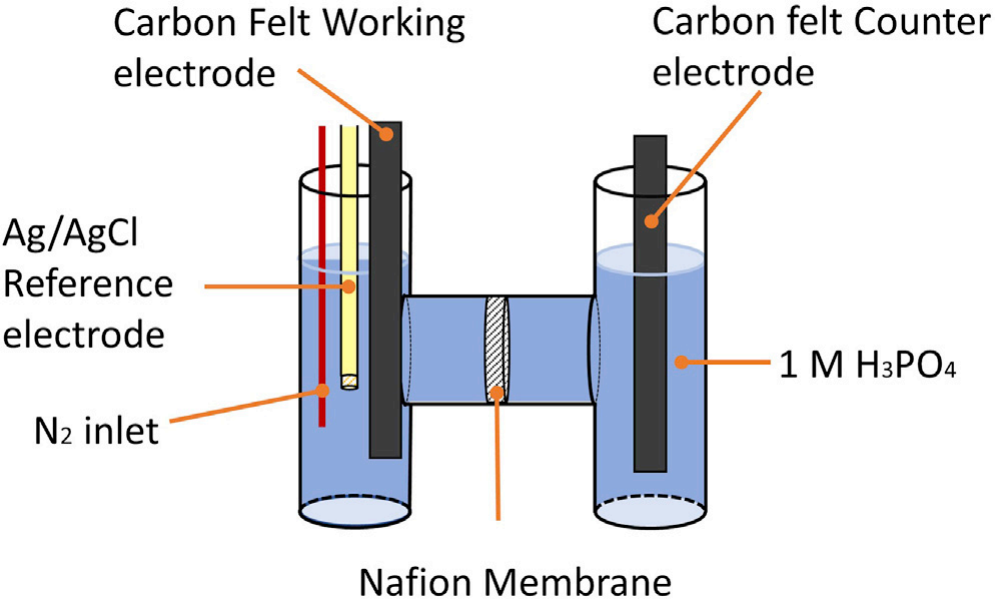
Representation of the two-compartment cell during the mediated electrolysis of nitrobenzene

**Scheme 1:**
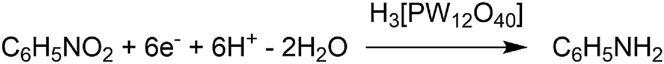
Overall reaction for the reduction of nitrobenzene via the use of the phosphotungstic acid redox mediator

This step describes the implementation of the two-compartment cell for the electrochemical reduction of nitrobenzene mediated by phosphotungstic acid as a redox mediator (see Figure 4 for a suggested cell set-up). The overall reaction is depicted in Scheme 1 below.

1. Add the following to the H-cell:
  a. If not already added, add 35 mL of 1 M aqueous phosphoric acid to each of the two compartments of the assembled cell.
  b. Add a stirring bar of the appropriate size to the working electrode compartment.
  c. Dissolve 0.280 g of phosphotungstic acid hydrate (10 mol% relative to nitrobenzene) in the solution in the working electrode compartment.
  d. Add 100 μL of nitrobenzene (9.74 × 10^–4^ mol) to the working electrode compartment (see troubleshooting 2).

***Note:*** Substituted nitroarenes have also been tested and are suitable for this protocol. If the starting material is significantly less water soluble than nitrobenzene, use half the molar amount and maintain the 10% mol ratio of mediator relative to the nitroarene.

***Note:*** For scale-up purposes, a larger custom-made electrochemical cell was used. The volume of each compartment was 110 mL and the active surface area of the working and counter electrodes was 3.2 × 4.5 cm. 10 mmol of nitrobenzene (1.23 g) was used with a 10 mol% ratio of phosphotungstic acid relative to nitrobenzene.

2. Clamp the cell over a stirrer plate so that the stirring bar in the working electrode compartment can rotate freely.
  a. Ensure that the cell is horizontal and level. Flat-bottomed cells were used in this protocol. Round-bottomed cells may also work, but this was not tested in this protocol.
3. Insert the components of the cell:
  a. Insert the working electrode and reference electrode into the working electrode compartment so that a geometrical surface area of 1.5 × 2 cm is submerged.
    i. Ensure that the crocodile clip holding the carbon felt electrode is not in contact with the electrolyte.
    ii. Ensure that the electrodes are not obstructing the motion of the stirrer bar.
    iii. Place the two electrodes as close as possible in space but ensure they do not touch.
    iv. Ensure the electrodes are fixed firmly in place (e.g., using Blu Tack or laboratory putty) and that they will not move during the reaction.
      ***Note:*** The same process can be performed using a two-electrode setup. In this case, there is no Ag/AgCl reference electrode, and the counter electrode serves as both the reference and counter electrodes. This can be achieved by connecting the potentiostat cable for the reference electrode to the counter electrode. A cell voltage of −2.2 V (with the electrode performing the nitrobenzene reduction being negative) is sufficient to pass appreciable current in the absence of large cell resistances. Pt mesh may be used as the counter electrode in such cases if the avoidance of oxidation of carbon felt is desired.
  b. Insert the N_2_ line into the working electrode compartment (e.g., using a long syringe needle).
    i. The tip of the syringe needle must be submerged about halfway into the electrolyte.
    ii. The syringe needle must not be in contact with either of the two electrodes.
    iii. The syringe needle must not obstruct the movement of the stirrer bar.
  c. Insert the counter electrode into the counter electrode compartment so that it is sufficiently submerged in the 1 M aqueous phosphoric acid solution. The distance between the working and counter electrode is about 11 cm in this particular set-up.
    i. Ensure the crocodile clip is not in contact with the electrolyte.
    ii. Ensure the electrode is fixed firmly in place and will not move during the reaction.
4. Connect the three electrodes to the appropriate cables of the BioLogic SP-150 potentiostat.
5. Stir the solution in the working electrode compartment at about 450–500 rpm.
6. Gently open the nitrogen gas valve to allow a gentle stream of nitrogen to flow through the syringe needle.
  a. Degas the working electrode compartment for a minimum of 20 min with stirring, prior to the electrolysis.
    ***Note:*** This will ensure that there is no dissolved oxygen in solution at the beginning of the reaction. The continuous flow of N_2_ during the reaction is necessary to avoid the presence of oxygen, which would otherwise lead to a decreased faradaic efficiency (see troubleshooting section for more details).
  b. The flow rate of the gas should be sufficient to see a constant stream of small bubbles, but no harsh degassing is suggested.
7. IR compensation (ZIR method) is enabled in sequence with chronoamperometry/ chronocoulometry (CA) to compensate for solution resistance with the following settings (Figure 5 shows how this is represented in the BioLogic potentiostat’s “EC-Lab” software).

**Figure 5:**
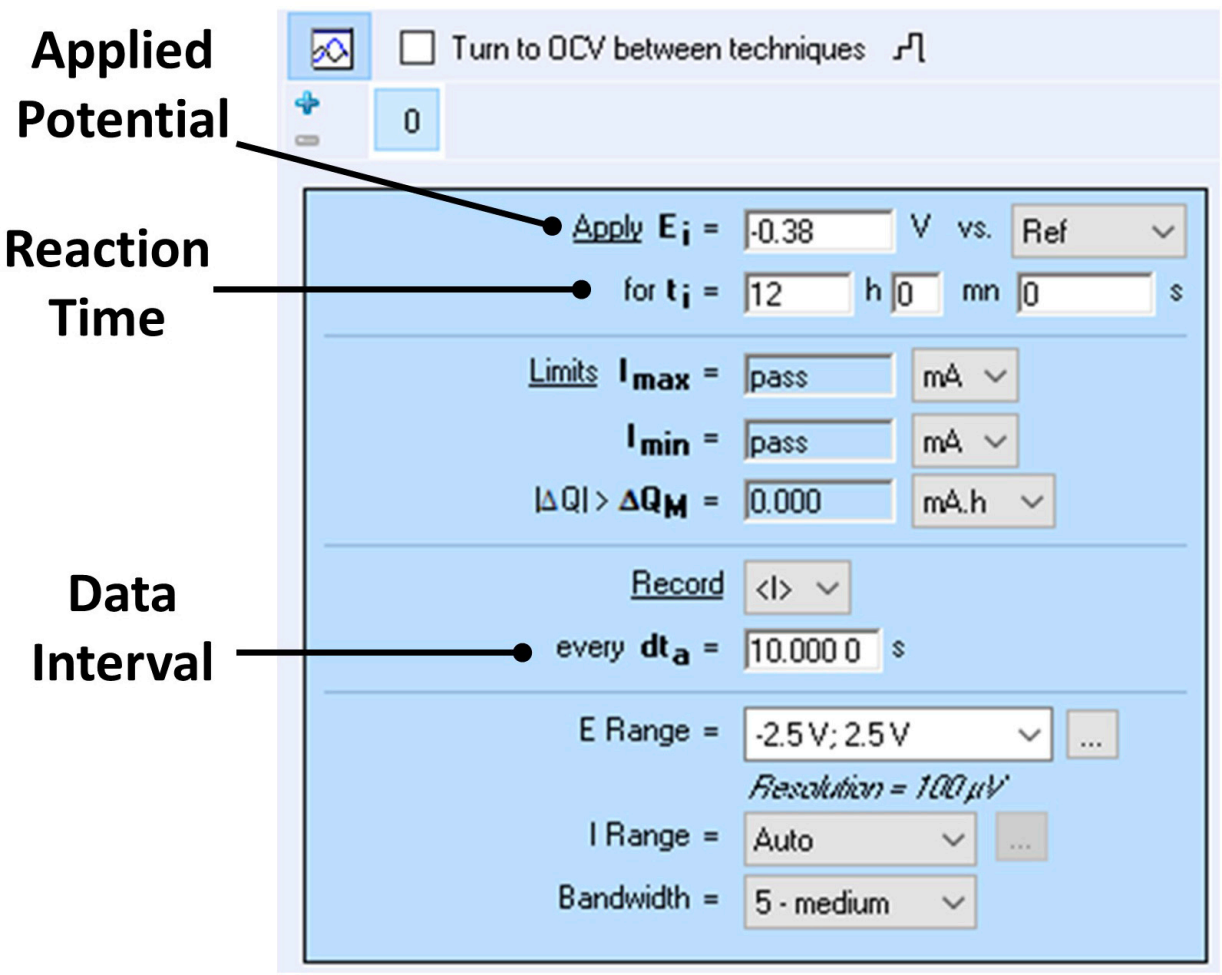
Chronoamperometry parameters used in the reduction of nitrobenzene in the EC-Lab software
8. Initiate the chronoamperometry experiment.
9. After completion, stop the electrolysis, the stirring, and remove the nitrogen line.

**CRITICAL:** It is critical to ensure that the H-cell is well positioned on the stirring plate and that the electrodes are firmly held in place and positioned in the solution in a way that firstly they do not touch each other, secondly that they do not disturb the stirrer bar and finally that the crocodile clip is not submerged in the solution, throughout the entire process.

The flow rate of the N_2_ gas should be controlled to minimize evaporation of the electrolyte and/or nitroarene substrate.

### Transfer the contents of the electrochemical cell

**Timing: 10 min**

This step describes the process of transferring the contents of the electrochemical cell into conical flasks for further analysis.

10. Transferring the contents of the cell.
  a. Take three 250 mL conical flasks and label them as organic phase (OP), working electrode compartment (WC) solution, and counter electrode compartment (CC) solution.
  b. The working electrode, stirring bar, and needle are thoroughly rinsed with deionized water into the WC flask.
  c. Subsequently, thoroughly rinse the same components using chloroform into the OP flask.
  d. Take the reference electrode and rinse thoroughly using deionized water into the WC flask. Return the reference electrode to its storage solution, 3 M NaCl.
  e. Empty the contents of the counter electrode compartment into the CC flask.
    i. Rinse the counter electrode and compartment using deionized water into the same flask.
    ii. Seal with parafilm and keep aside, until the analysis of the reaction product is complete.
  f. Empty the contents of the working electrode compartment into the WC flask.
    i. Rinse the working electrode compartment of the cell with deionized water.
  g. Then rinse the working electrode compartment with chloroform into the OP flask.
  h. At this stage, the working and counter electrode carbon felt electrodes can be disposed of.

### pH adjustment of the working electrode compartment solution

**Timing: 5 min**

This step describes the process of pH analysis and adjustment of the working electrode compartment solution (WC) prior to the organic extraction.

11. Adjust the pH of the WC well above the p*K*_a_ value of the desired product aniline (e.g., well above 5.6) to deprotonate any protonated aniline species.
  a. Calibrate the pH meter daily using calibration solutions for more accurate readings.
  b. Place a pH probe into the aqueous phase (WC) flask.
  c. Slowly add 1 M sodium hydroxide solution while stirring the solution in the WC flask. Stop when the pH meter generates a stable reading at about 7.
    ***Note:*** When/if different nitroarenes are used, adjust the aqueous phase to a pH that is at least 1.0 unit higher than the p*K*_a_ of the desired product in order to ensure an adequate extent of neutralization prior to extraction into the organic solvent.
  d. Rinse the pH probe sparingly with deionized water into the working electrode compartment conical flask.

### Organic extraction

**Timing: 20 min**

This step describes the process of the organic extraction of aniline and other potential intermediates from the aqueous solution.

12. Extraction of the aqueous phase into the organic solvent.
  a. Transfer the contents of the WC flask into a 250 mL separating funnel.
    i. Rinse the flask thoroughly with deionized water and add the washings to the separating funnel.
    ii. Rinse the flask thoroughly with chloroform and add the washings to the separating funnel.
  b. Transfer the contents of the OP flask to the same separating funnel and rinse the OP flask three times with chloroform, adding the washings to the separating funnel.
  c. Perform three extractions of the solution in the separating funnel with chloroform and collect the organic phases in the OP flask.
    i. Use around 50 mL of chloroform for each extraction.
  d. Collect the aqueous phase from the separating funnel in a clean conical flask and set it aside until the product identification is complete.
13. Wash the combined organic extracts.
  a. Place the combined organic extracts back into a clean separating funnel and wash three times with deionized water (20 mL).
    ***Note:*** This is to remove any water-soluble organic species left from the previous steps.
  b. Drain the washed combined organic extracts into a clean conical flask.
  c. Keep the aqueous washings aside until full analysis of the organic extract.
14. Dry the organic extract using magnesium sulfate.
  a. Add magnesium sulfate slowly to the organic extract flask while gyrating until any additional magnesium sulfate remains free flowing.
    ***Note:*** This will remove any water traces left in the organic extract.
  b. Filter the magnesium sulfate from the organic solution.
    i. Set up a clean conical flask with a funnel and fitted filter paper.
    ii. Pour the contents of the organic phase flask into the funnel and filter under gravity.
    iii. Wash the organic extract conical flask with chloroform and pour these washings through the funnel.
    iv. Once all the organic solution has fully filtered through, rinse the magnesium sulfate left on the filter paper with small amounts of chloroform.

### Organic extract analysis

**Timing: 45–60 min**

This step describes the preparation of the NMR sample for the analysis of the synthesized product(s).

15. Concentrate the organic phase.
  a. Weigh a clean and dry 250 mL or 500 mL round bottom flask.
  b. Transfer the contents of the filtered chloroform conical flask into the round bottom flask. Rinse the conical flask with chloroform and add the rinsing to the round bottom flask.
  c. Place the flask on a rotary evaporator and use the following settings:
    i. Water bath temperature: 45°C.
    ii. Set the pressure at 450 mbar. Note, slowly reduce the pressure to prevent rapid suction of contents into the trap or condenser.
  d. Once all the chloroform is removed, decrease the pressure to give a high vacuum for at least 30 min.
  e. Remove the flask from the rotary evaporator and allow it to cool down to 20°C–25°C.
  f. Reweigh the flask and make a note of the gross yield.
16. Prepare the NMR sample.
  a. Redissolve the contents of the round bottom flask in 0.5 mL of deuterated chloroform.
  b. Remove the contents of the flask using a syringe or pipette and transfer to an appropriately labeled NMR tube.
  c. Obtain the ^1^H NMR spectrum.

***Note:*** Common intermediates of the reduction of nitrobenzene are *N*-phenylhydroxylamine and azoxybenzene which may appear in the NMR spectrum and should be considered as undesirable side products for conversion and selectivity calculations.

**Pause point:** The electrolysis reaction can be paused and/or stopped at any point, although depending on the time that has elapsed, conversion to aniline may not be complete and intermediates may be present in the reaction mixture. For example, after 1 h of reaction under the conditions specified above, the reaction mixture will consist of around 80% of unreacted nitrobenzene and 12%–15% aniline, with traces of *N*-phenylhydroxylamine and azoxybenzene also present.

## Expected outcomes

**Figure 6:**
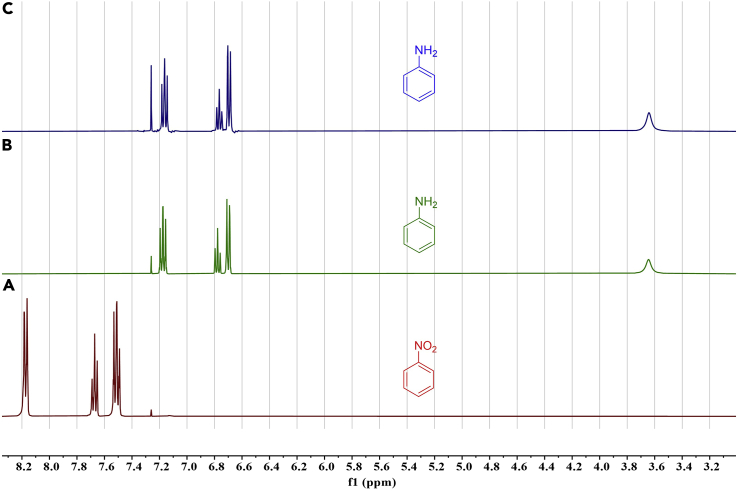
Reduction of nitrobenzene (A–C) ^1^H NMR spectra as follows; the nitrobenzene starting material (A), a sample of pure aniline used as a standard (B), and the spectrum of the electrocatalytic reaction medium after extraction and concentration (C). All spectra were obtained in CDCl_3_. Spectra B and C are essentially indistinguishable, showing clean conversion to aniline.

The expected isolated yield for nitrobenzene should be above 80% with suitable tolerance for losses during the extraction process. At the same time, the conversion and selectivity for the reduction of nitrobenzene in the presence of 10 mol% of the mediator was >99%. Figure 6 illustrates stacked ^1^H NMR spectra showing the successful formation of aniline, and from which the selectivity and conversion can be obtained.

The Faradaic efficiency, which is a metric of the efficiency of the electrochemical process, was 95% and the current density during the first 2.5 h of the reaction was greater than 5 mA/cm^2^ (based on the geometric area of the working electrode submerged in the electrolyte). Please note that when using different nitroarene starting materials, these values may vary.

## Quantification and statistical analysis

### NMR analysis

1. Calculate conversion, selectivity, and isolated yield using the NMR software.
  a. Identify and assign the expected product peaks in the ^1^H NMR spectrum.
    i. Identify and assign all the rest of the ^1^H NMR peaks in the spectrum, if there are any.
  b. Integrate the peaks of all the species present in the spectrum.
    i. Compare the integrals of the peaks for the various products to those of the starting materials to calculate the conversion, making sure that peaks corresponding to the equivalent number of protons in the various possible structures are compared. This may require the practitioner to first identify the species present and their characteristic peak patterns.
    ii. Compare the integrals of the peaks of the desired product to the sum of those for the intermediates and starting materials (if applicable) to calculate the selectivity towards the desired product, making sure that peaks corresponding to the equivalent number of protons are compared.
    iii. Use gross yield, conversion, and selectivity to calculate the isolated yield.

### Chronoamperometric analysis

2. Using the information taken from the software of the potentiostat, calculate the average current density and Faradaic efficiency.
  a. Use the current reading over the first (e.g.) 2.5 h of the reaction and divide it by the geometric surface area of the working electrode submerged in the electrolyte to calculate the average current density in mA/cm^2^ over that time period. More meaningful average current densities will be obtained by selecting a time period over which the current is roughly linear.
  b. Use the recorded charge passed during the reaction and the isolated yield of the desired product to calculate the Faradaic efficiency of the reaction.
    i. From the Faraday’s law of electrolysis (Equation 1), calculate the maximum theoretical moles of the desired product that could have been synthesized if all of the charge passed had gone towards making that product.
      (Equation 1) $$\begin{array}{l} Q=mnF \end{array}$$
      Where *Q* is the charge passed in C, *m* is the number of moles of the electroactive species in question, *n* is the number of electrons transferred per mole of electroactive species and *F* is the Faraday constant and is equal to 96,485 C mol^−1^. For conversion of nitrobenzene to aniline, *n* = 6.
    ii. Calculate the actual number of moles of product obtained from the isolated mass of the product.
    iii. Use Equation 2 to calculate the Faradaic efficiency (%) (FE):

(Equation 2) $$\begin{array}{l} FE\left(\%\right)=\frac{actual\;moles\;obtained}{theoretical\;moles}\;\times 100 \end{array}$$

## Limitations

Due to the small amounts of starting materials and products, the yield of the electrochemical process can vary because of potential losses during the organic extraction process. It is important to take extra care during these stages to ensure that all the material is transferred when moving solutions from one container to another. Also, ensure the H-cell is thoroughly rinsed as the shape of the cell can cause material to be trapped in joints that are hard to reach. Moreover, some nitroarene substrates have low to no water solubility. Whilst this does not seem to be a strict barrier to successful electro-reduction, the speed of the reaction process is slowed for substrates that are especially insoluble. Finally, it is worth mentioning that the electro-reduction process works much more efficiently under an inert atmosphere such as nitrogen or argon gas. Whilst the reaction still works highly selectively under air, the Faradaic yield is diminished as the reduced redox mediator can be re-oxidized by oxygen in the air, rather than by reaction with the nitrobenzene substrate.

## Troubleshooting

### Problem 1

Leaking cell.

There is the potential issue of cell leakage (e.g., through the join between the two compartments). Please refer to electrochemical cell assembly section, step 5.b.

### Potential solution

• Use Teflon tape to seal joints.
• Use a new sealing ring if there is a noticeable deformation.
• Ensure components are tightly threaded together.
• Make sure that the two-compartment cell is level with the surface of the stirring plate prior to electrolysis.

### Problem 2

Low solubility of starting material.

Some nitroarenes have low to no solubility in the aqueous electrolyte. However, reaction with the mediator producing the desired product is still possible. The following solutions do not, therefore, necessarily improve the obtained conversion/selectivity, but they may reduce the reaction time required. Please refer to the electrolysis section, step 1.d.

### Potential solution

• Mildly sonicate the working electrode compartment solution, containing the undissolved substrate prior to electrolysis, to encourage more substrate to dissolve.
• Heat up the working electrode compartment solution to about 60°C (with stirring, and/or sonication) for about 10 min to encourage more substrate to dissolve.

### Problem 3

Presence of oxygen during the electrolysis.

The reaction of oxygen with the reduced mediator re-oxidizes the mediator and so results in decreased Faradaic efficiency. Based on our previous studies, in the presence of oxygen the Faradaic efficiency decreased to 73%. The half-life for the re-oxidation of the reduced polyoxometalate redox mediator in air was found to be about 30 min (Stergiou and Symes, 2022a). Please refer to the electrolysis section, step 3.b.

### Potential solution

N_2_ gas should be purged through the working electrode compartment of the H-cell throughout the electrolysis.

### Problem 4

Short circuit.

The necessity of purging N_2_ through the working electrode compartment of the H-cell during the electrolysis raises the prospect that the N_2_ inlet (in this case a metallic needle) could touch the working electrode surface or the crocodile clip that is used to connect the potentiostat with the working electrode, giving a short circuit. Please refer to the electrolysis section, step 3.b.

### Potential solution

The N_2_ inlet (metallic needle) should be firmly fixed into the working electrode compartment using Blu Tack or a similar laboratory putty to ensure that the needle is in not in contact with the working electrode during the electrolysis. If this is not possible, then a non-electrically conductive N_2_ inlet should be considered.

## Resource availability

### Lead contact

Further information and requests for resources and reagents should be directed to and will be fulfilled by the lead contact, Mark Symes (mark.symes@glasgow.ac.uk).

### Materials availability

This study did not generate new unique reagents.

## Data Availability

Original/source data for Figure 5 and Table 1 in the paper are available at https://doi.org/10.5525/gla.researchdata.1279.

**Table 1:** Summary of the example information after NMR and chronoamperometry interpretation

Average current density (mA/cm^2^)	Nitrobenzene conversion (%)	Aniline selectivity (%)	Azoxybenzene selectivity (%)	Phenylhydroxyl-amine selectivity (%)	Nitrosobenzene selectivity (%)	Isolated yield of aniline (%)	Faradaic efficiency (%)
–5.31	>99	>99	0	0	0	**90±2**	95 Summary of the example information after NMR and chronoamperometry interpretation
